# A General Numerical Error Compensation Method for NLFM Signal in SAR System Based on Non-Start–Stop Model

**DOI:** 10.3390/s25092770

**Published:** 2025-04-27

**Authors:** Gui Wang, Heng Zhang, Bo Li, Weidong Yu

**Affiliations:** 1 Department of Space Microwave Remote Sensing System, Aerospace Information Research Institute, Chinese Academy of Sciences, Beijing 100094, China; wanggui16@mails.ucas.ac.cn (G.W.); zhangheng@aircas.ac.cn (H.Z.); libo202@mails.ucas.edu.cn (B.L.); 2 School of Electronic, Electrical and Communication Engineering, University of Chinese Academy of Sciences, Beijing 100049, China

**Keywords:** synthetic aperture radar, nonlinear frequency modulated signal, non-start–stop model, numerical error compensation, imaging processing

## Abstract

Nonlinear frequency modulated (NLFM) signals can be used to enhance the resolution, anti-jamming capability, and imaging quality of synthetic aperture radar (SAR) systems through optimized design, demonstrating substantial application potential. However, in a SAR system using NLFM signals, the non-start–stop effect, caused by the continuous motion of the platform during pulse transmission and reception, introduces significant errors, resulting in target defocusing. To tackle this problem, this paper proposes a general numerical error compensation method dedicated to NLFM signals. First, the error model is correspondingly derived from the non-start–stop assumption. Then, a phase compensation method is designed through numerical calculations. Simulation experiments are performed to validate the effectiveness of the proposed method. This method provides a robust error compensation framework for high-resolution SAR systems using NLFM signals.

## 1. Introduction

Synthetic aperture radar (SAR) is an active microwave remote sensing imaging system capable of all-weather and all-day operation, playing a crucial role in military reconnaissance, topographic mapping, and environmental monitoring. Over the past few decades, SAR technology has advanced rapidly, achieving high resolution, high coverage, multi-polarization, and multi-band capabilities [1,2,3].

High-resolution SAR imaging facilitates enhanced target characterization through the acquisition of extensive spatial details, rendering it highly advantageous for defense-related reconnaissance applications [4]. These advantages have driven extensive research efforts in advanced SAR systems. However, emerging imaging techniques such as spotlight and sliding spotlight modes present complex challenges, including precise motion compensation, spectrum aliasing processing, start–stop approximation correction, and computational efficiency optimization [5,6,7,8]. While conventional start–stop models assuming static radar positions during pulse transmission/reception remain effective for conventional low-resolution SAR systems [9], their limitations become apparent in high-speed platform deployments requiring ultra-fine resolution. The inherent approximation errors in this traditional approach may critically degrade imaging performance under demanding operational conditions [5,6,7]. This necessitates the development of continuous-motion models that account for platform displacement within pulse repetition intervals. The non-start–stop model, which explicitly addresses radar movement during both signal transmission and reception phases, has consequently become essential for modern high-performance SAR implementations [7,8,9,10].

Conventional SAR implementations predominantly utilize linear frequency modulated (LFM) signals, with substantial research dedicated to quantifying start–stop approximation errors. Prats Iraola P. established an analytical framework decomposing temporal distortions into fast-time and slow-time components, validated through TanDEM-X mission data analysis [5]. Comparative studies between classical and revised echo models further quantify these approximation errors, with numerical simulations demonstrating their critical impact under operational constraints [6]. To address these limitations, innovative processing architectures have emerged: A hybrid approach integrating conventional two-step processing with backprojection principles was experimentally verified using Gaofen-3 satellite observations [7], while enhanced chirp scaling algorithms incorporating continuous platform motion models demonstrate measurable improvements in computational efficiency and geometric fidelity [8]. Recent advances extend to spaceborne implementations, where continuous tangent motion compensation enables 0.21 m resolution imaging across 225 km^2^ coverage areas [10]. Bistatic SAR configurations have also benefited from generalized echo formulations accounting for dynamic platform trajectories during signal propagation [11]. Notably, existing theoretical frameworks predominantly address LFM signal scenarios, revealing a significant research gap regarding nonlinear frequency modulated (NLFM) signal applications. Preliminary investigations into Doppler shift effects on NLFM-SAR configurations propose central frequency compensation techniques [12], yet these solutions prove inadequate for high-resolution implementations requiring variant error correction. This limitation underscores the imperative for developing comprehensive compensation methodologies tailored to advanced NLFM-SAR operational requirements.

NLFM signals have emerged as a pivotal innovation in SAR, demonstrating exceptional efficacy in suppressing sidelobes without signal-to-noise (SNR) loss [13,14,15,16,17,18,19,20,21,22,23,24,25,26,27]. The first airborne SAR validation of NLFM signal is demonstrated in [13], where waveform optimization significantly enhanced imaging precision. Subsequent advancements, such as the operator optimization method to design advanced NLFM signals, achieved a 1.29 dB enhancement in target contrast compared with traditional LFM systems [15]. These studies have not only improved SAR image quality but also expanded its applicability in unmanned aerial vehicle and SAR missions [18]. Notably, the integration of linear interpolation-based NLFM signal generators aboard the LuTan-1 satellite [20] exemplifies the transition from theoretical frameworks to operational deployments. A genetic algorithm-optimized NLFM signal, characterized by adaptive piecewise chirp-rate modulation, is proposed in [22], which balances sidelobe reduction with reconfigurability for diverse SAR imaging scenarios. These innovations underscore NLFM signal’s transformative role in high-fidelity remote sensing. To fully harness its capabilities, future work must prioritize adaptive compensation strategies tailored to high-resolution SAR systems.

To this end, significant errors introduced by the non-start–stop model in the SAR system using NLFM signals should be studied. In this paper, a general numerical error compensation method for NLFM signal in SAR system based on the non-start–stop model is proposed. In addition, simulation experiments are also performed to verify its effectiveness. The remainder of this paper is organized as follows. In Section 2, the LFM and NLFM signals’ properties are introduced and compared in detail. Then, the non-start–stop model is described in Section 3. The error is derived from the non-start–stop model and numerical calculation compensation in Section 4. In Section 5, the numerical experiment is performed to verify the effectiveness of the proposed algorithm. Finally, the conclusion is drawn in Section 6.

## 2. LFM and NLFM Signal Model and Property

### 2.1. LFM Signal

In SAR systems, the wide-duration pulse with frequency modulation is transmitted, and compression is applied to achieve the desired resolution. For the LFM signal, the waveform can be expressed as [9]:

(1) $$s(t)=\mathrm{rect}(\frac{t}{T})\exp(j\pi Kt^{2})$$

where t represents time, T is the pulse duration, and K denotes the chirp rate. rect(⋅) denotes rectangular function. The spectrum of the LFM signal s(t) is approximated as:

(2) $$s(f)=\mathrm{rect}(\frac{f}{\left|K\right|T})\exp(-j\pi \frac{f^{2}}{K})$$

The received echo from a target with a time delay $t_{0}$ is:

(3) $$s_{r}(t)=\mathrm{rect}(\frac{t-t_{0}}{T})\exp(j\pi K{\left(t-t_{0}\right)}^{2})$$

Setting $t_{0}=0$, the matched filter h(t) is the time-reversed complex conjugate of: s(t)

(4) $$h(t)=s^{*}(-t)=\mathrm{rect}(\frac{t}{T})\exp(-j\pi Kt^{2})$$

The output of the matched filter after convolution approximates a sinc function. The peak sidelobe ratio (PSLR) is approximately −13.26 dB, consistent with the Fourier transform of a rectangular window. The impulse response width (IRW) (i.e., 3 dB resolution) is defined as the interval between two −3 dB points in the compressed pulse. In the time domain, the resolution of the LFM signal is given by [9]:

(5) $$\mathrm{IRW}=\frac{0.886}{\left|K\right|T}$$

### 2.2. NLFM Signal

The key distinction between NLFM and LFM signals lies in the nonlinear relationship between instantaneous frequency and time. For the NLFM signal, the chirp rate K(t) becomes time-dependent. Its analytical expression is challenging to derive but can be constructed using the principle of stationary phase (POSP). According to POSP, for a signal with a large time-bandwidth product, the power spectral density (PSD) at a certain frequency is inversely proportional to the frequency modulation rate of its time-domain signal at that frequency [13]. Therefore, by designing a frequency modulation signal with a specific modulation rate at a particular frequency, the requirements of the PSD can be met. Suppose the phase of the NLFM signal is ϕ(t), and using the expression for the LFM signal, the expression for the NLFM signal can be written as [13]:

(6) $$s(t)=\mathrm{rect}(\frac{t}{T})\exp(j\phi (t))$$

The instantaneous frequency $f\left(t\right)$ is the derivative of the phase ϕ(t), expressed as:

(7) $$f\left(t\right)=\frac{1}{2\pi }\cdot \frac{d\phi (t)}{dt}$$

where $f\left(t\right)$ has units of Hz and is a function of time t. The chirp rate K(t), defined as the derivative of $f\left(t\right)$, can be derived as [25]:

(8) $$K(t)=\frac{df\left(t\right)}{dt}=\frac{1}{2\pi }\frac{d\phi^{2}(t)}{dt^{2}}$$

The PSD amplitude P(f) is inversely proportional to K(t), expressed as:

(9) $$P(f)=\frac{a}{K(t)}=2\pi \cdot \frac{a}{\phi^{\prime \prime }(t)}=a\cdot \frac{dt}{df}$$

where a is a constant and can be calculated by $a=1/T_{p}\int_{-B_{r}/2}^{B_{r}/2}P(f)df$. By defining t=T(f) (time as a function of instantaneous frequency), T(f) can be derived as follows:

(10) $$T(f)=\int_{0}^{f}T^{\prime }(f)df=\int_{0}^{f}\frac{P(f)}{a}df$$

In practice, the coefficients of the function T(f) can be obtained through polynomial curve fitting, and the discrete values can be integrated to obtain the phase $\phi_{F}(f)$ in the frequency domain. Therefore, $\phi_{F}(f)$ can be expressed as:

(11) $$\phi_{F}(f)=2\pi (\frac{b_{1}}{2}f^{2}+\frac{b_{2}}{3}f^{3}+\frac{b_{3}}{4}f^{4}+\cdots )$$

where $b_{i}(i=1,2,\cdots )$ denote the corresponding coefficient. The number $b_{i}$ can be ascertained by means of the residual error.

The inverse of T(f) is the instantaneous frequency as a function of time $f(t)=T^{-1}(f)$. As a result, the NLFM signal can be written as [13]:

(12) $$s(t)=\mathrm{rect}(\frac{t}{T})\exp\left(j2\pi \left(\int_{0}^{t}f\left(\tau \right)d\tau \right)\right)$$

There are many weighting functions that can be used to obtain a low sidelobe autocorrelation function. Taking the Taylor weighting function as an example for constructing the NLFM signal, the PSD amplitude function is [13]:

(13) $$P(f)=\mathrm{rect}(\frac{f}{B_{r}})\left[1+2\sum_{m=1}^{\bar{n}-1}F_{m}\cos\left(\frac{2\pi mf}{B_{r}}\right)\right]$$

where $F_{m}=F(m,\xi ,\bar{n})$ is the coefficient of the *m*-th order parameter, ξ represents the peak sidelobe level ratio, and $\bar{n}$ represents the number of sidelobes with amplitudes similar to the main lobe. Using Equation (13), we can obtain [13]:

(14) $$T(f)=\mathrm{rect}(\frac{f}{B_{r}})\left[\frac{T}{B_{r}}f+\sum_{m=1}^{\bar{n}-1}\frac{F_{m}T}{\pi m}\cos\left(\frac{2\pi mf}{B_{r}}\right)\right]$$

However, the inverse function expression is difficult to obtain. In a similar way, the coefficients of the function f(t) can be obtained through polynomial curve fitting, and the discrete values can be integrated to obtain ϕ(t). Therefore, ϕ(t) can be expressed as:

(15) $$\phi (t)=2\pi (\frac{d_{1}}{2}t^{2}+\frac{d_{2}}{3}t^{3}+\frac{d_{3}}{4}t^{4}+\cdots )$$

where $d_{i}(i=1,2,\cdots )$ denote the corresponding coefficient.

### 2.3. Comparison Between LFM and NLFM

**Figure 1:**
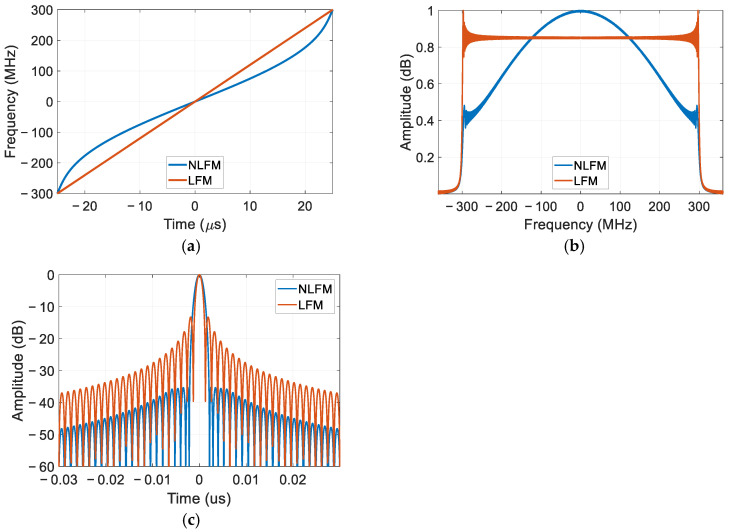
LFM and NLFM signals. (**a**) Frequency versus time; (**b**) Normalized PSD; (**c**) Compression result.

In the subsequent analysis, an LFM signal is compared with NLFM. The instantaneous frequency is depicted in Figure 1a. This results in the obtained NLFM signal exhibiting a spectrum resembling that of the PSD in Figure 1b, thereby substantiating the validity of the constructed NLFM signal. The compressed signal can be seen in Figure 1c. It can be seen that the low sidelobe of the NLFM signal is obtained compared with the LFM signal.

## 3. SAR Echo Signal

### 3.1. Start–Stop Model

**Figure 2:**
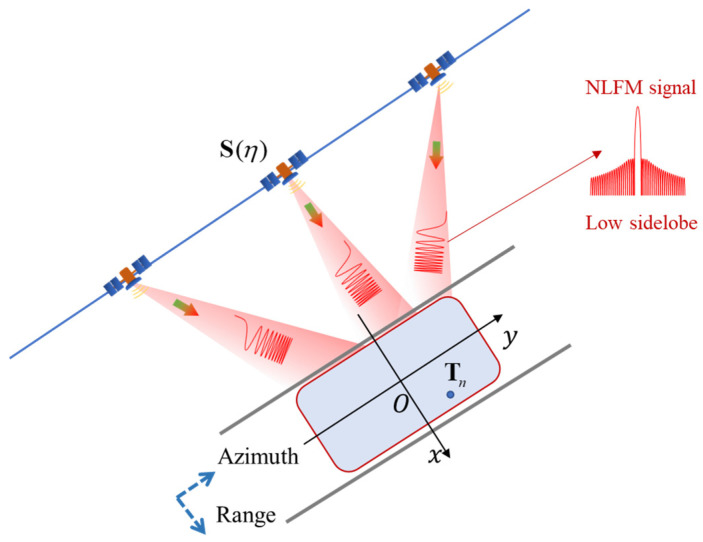
SAR imaging configuration with NLFM signal.

Establish a Cartesian coordinate system with the center of the scene as the origin, where the direction x is along the ground distance direction, the direction y is along the square position direction, and the direction z is the height direction (as shown in Figure 2).

Assuming the position of the target in the scene is $T_{n}=(x_{n},y_{n},z_{n})$, where *n* denotes the number of the target. In azimuth time η, the satellite position is $S(\eta )=(x_{\eta },y_{\eta },z_{\eta })$, the echo delay t(η) can be derived as [8,9]:

(16) $$t(\eta )=2R(\eta )/c=2\|S(\eta )-T_{n}\|/c$$

where c is the speed of light. The SAR echo based on the start–stop model can be represented as [9]:

(17) $$S_{1}(t,\eta )=w_{a}(\eta )w_{r}\left(t-t(\eta )\right)\exp\left\{j\phi \left(t-t(\eta )\right)\right\}\exp\left\{-j2\pi f_{0}t(\eta )\right\}$$

where $w_{a}(\eta )$ represents the azimuth antenna pattern and $f_{0}$ is carrier frequency. To simplify the analysis, $w_{a}(\eta )$ and $w_{r}(t)$ in Equation (17) are ignored in the subsequent analysis. $\phi \left(t\right)$ denotes the modulated signal (can be LFM or NLFM signals).

### 3.2. Non-Start–Stop Model

Under the non-start–stop model, the target delay $t_{d}$ is a function of azimuth time η and range time t. $R(\eta +t_{s})=\|S(\eta +t_{s})-T_{n}\|$ and $R(\eta +t)=\|S(\eta +t)-T_{n}\|$ denote the transmission and reception distances between the *n*-th target and satellite, respectively [8]. $\left(\eta +t_{s}\right)$ represents the signal transmission time. Suppose the velocity of the satellite at time η is V(η) (assumed to remain constant within a PRI), $t_{d}$ can be expressed as [8]:

(18) $$t_{d}=t_{d,n}(\eta )+\alpha_{n}(\eta )t$$

where

(19) $$t_{d,n}(\eta )=\frac{2R(\eta )}{c+\|V(\eta )\|\cos\theta_{n}(\eta )},\;\alpha_{n}(\eta )=\frac{2\|V(\eta )\|\cos\theta_{n}(\eta )}{c+\|V(\eta )\|\cos\theta_{n}(\eta )}$$

where $\theta_{n}(\eta )$ can be represented as [8]:

(20) $$\theta_{n}(\eta )=\arccos\left(\frac{V(\eta )\cdot (S(\eta )-T_{n})}{\left\|V(\eta )\|\;\|S(\eta )-T_{n})\right\|}\right)$$

As a result, the SAR echo based on the non-start–stop model can be written as [8]:

(21) $$\begin{array}{l} S_{2}(t,\eta )=\exp\left\{j\phi \left(t-t_{d}\right)\right\}\exp\left\{-j2\pi f_{0}t_{d}\right\}\\ \;\;\;=\exp\left\{j\phi \left(\left(1-\alpha_{n}(\eta ))t-t_{d,n}(\eta \right)\right)\right\}\exp\left\{-j2\pi f_{0}(t_{d,n}(\eta )+\alpha_{n}(\eta )t)\right\} \end{array}$$

A detailed comparison between SAR echoes based on the start–stop model and the non-start–stop model has been discussed in [8].

## 4. Error Analysis and Numerical Compensation Method

### 4.1. Error Analysis

Suppose the Fourier transform of a function f(t) is F(ω), which can be written as:

(22) $$F(\omega )=\int_{-\infty }^{\infty }f(t)e^{-j\omega t}dt=F\{f(t)\}$$

Then Fourier transform of the function $f(at)e^{-j\omega_{0}t}$ can be expressed as [28]:

(23) $$F\{f(at)e^{-j\omega_{0}t}\}=\frac{1}{\left|a\right|}F\left(\frac{\omega +\omega_{0}}{a}\right)$$

As a result, suppose the Fourier transform of $S_{1}(t,\eta )$ and $S_{2}(t,\eta )$ in range direction are $S_{1}(f_{t},\eta )$ and $S_{2}(f_{t},\eta )$, respectively, then the relationship between the $S_{1}(f_{t},\eta )$ and $S_{2}(f_{t},\eta )$ can be written as:

(24) $$S_{2}(f_{t},\eta )=\frac{1}{\left|1-\alpha_{n}(\eta )\right|}S_{1}(\frac{f_{t}+f_{0}\alpha_{n}(\eta )}{1-\alpha_{n}(\eta )},\eta )$$

In general, ‖V(η)‖≈7000 m/s for low-orbit SAR satellite SAR. Due to the fact that ‖V(η)‖≪c = $3\cdot 10^{8}$ m/s, $\alpha_{n}(\eta )$ can be approximated as:

(25) $$\alpha_{n}(\eta )=\frac{2\|V(\eta )\|\cos\theta_{n}(\eta )}{c+\|V(\eta )\|\cos\theta_{n}(\eta )}\approx \frac{2\|V(\eta )\|\cos\theta_{n}(\eta )}{c}=\frac{f_{\eta }}{f_{0}}$$

We find that $f_{t}+f_{0}\alpha_{n}(\eta )$ can be derived as:

(26) $$f_{t}+f_{0}\alpha_{n}(\eta )\approx f_{t}+\frac{2\|V(\eta )\|\cos\theta_{n}(\eta )}{c}f_{0}=f_{t}+f_{\eta }$$

In addition,

(27) $$\alpha_{n}(\eta )\approx \frac{2\|V(\eta )\|\cos\theta_{n}(\eta )}{c}\leq \frac{2\|V(\eta )\|}{c}\approx \frac{2\cdot 7000}{3\cdot 10^{8}}=4.667\cdot 10^{-5}\ll 1$$

Therefore, $\alpha_{n}(\eta )$ is very small, and the amplitude modulation $\frac{1}{\left|1-\alpha_{n}(\eta )\right|}$ is almost negligible for target focusing; as a result:

(28) $$S_{2}(f_{t},\eta )=\frac{1}{\left|1-\alpha_{n}(\eta )\right|}S_{1}(\frac{f_{t}+f_{0}\alpha_{n}(\eta )}{1-\alpha_{n}(\eta )},\eta )\approx S_{1}(\frac{f_{t}+f_{0}\alpha_{n}(\eta )}{1-\alpha_{n}(\eta )},\eta )=S_{1}(\frac{f_{t}+f_{\eta }}{1-f_{\eta }/f_{0}},\eta )$$

From a signal processing perspective, Equation (28) can be interpreted as follows: the satellite has been in motion during the transmission and reception of the signal, resulting in a frequency deviation and scale transformation of the signal. The frequency shift is equivalent to the Doppler frequency $f_{\eta }$, and the scale shift is related to the Doppler frequency and the carrier frequency.

At this point, in order to transform $S_{2}(f_{t},\eta )$ to $S_{1}(f_{t},\eta )$ then the traditional algorithm can be performed to obtain the imaging result, the phase error should be compensated as follows:

(29) $$\theta_{error}=\theta_{1}(f_{t},\eta )-\theta_{2}(f_{t},\eta )=\theta_{1}(f_{t},\eta )-\theta_{1}(\frac{f_{t}+f_{\eta }}{1-f_{\eta }/f_{0}},\eta )=\phi_{F}(f_{t})-\phi_{F}(\frac{f_{t}+f_{\eta }}{1-f_{\eta }/f_{0}})$$

where $\theta_{1}(f_{t},f_{\eta })$ and $\theta_{2}(f_{t},f_{\eta })$ denote the phases of $S_{1}(f_{t},\eta )$ and $S_{2}(f_{t},\eta )$, respectively. Az azimuth time η, the corresponding Doppler frequency is $f_{\eta }$. Consequently, the compensation phase can be performed in the 2D frequency domain.

An LFM signal can be regarded as a special NLFM signal, and its phase can be derived as:

(30) $$\phi_{F}(f_{t})=-\frac{\pi f_{t}^{2}}{K}$$

Therefore, the compensation phase is:

(31) $$\theta_{error}=\phi_{F}(f_{t})-\phi_{F}(\frac{f_{t}+f_{\eta }}{1-f_{\eta }/f_{0}})\approx \frac{2\pi f_{t}f_{\eta }}{K}$$

which is the same as the analysis in [5,6,7].

For the NLFM signal, the phase $\theta_{error}$ can be expressed as follows:

(32) $$\begin{array}{l} \theta_{error}=\phi_{F}(f_{t})-\phi_{F}(\frac{f_{t}+f_{\eta }}{1-f_{\eta }/f_{0}})\\ \;\;=2\pi (\frac{b_{1}}{2}f_{t}^{2}+\frac{b_{2}}{3}f_{t}^{3}+\cdots )-2\pi [\frac{b_{1}}{2}(\frac{f_{t}+f_{\eta }}{1-f_{\eta }/f_{0}}{)}^{2}+\frac{b_{2}}{3}(\frac{f_{t}+f_{\eta }}{1-f_{\eta }/f_{0}}{)}^{3}+\cdots ] \end{array}$$

**Figure 3:**
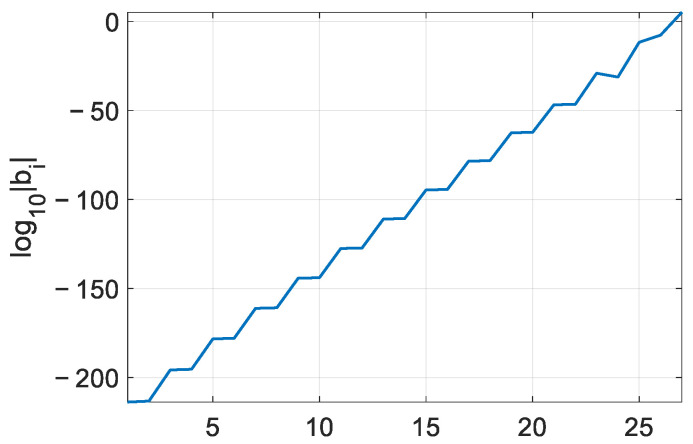
The value of $b_{i}$.

The compensation phase can be obtained through a numerical calculation using Equation (32) for the NFLM signal. Here, some examples are illustrated to analyze the error. The simulation parameters can be seen in Table 1. Here, a 26th order coefficient is used. The values of $b_{1}$~$b_{26}$ can be seen in Figure 3.

**Figure 4:**
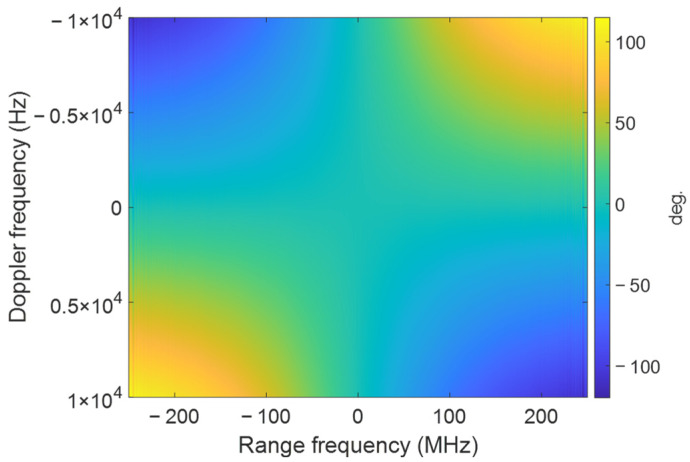
The compensation phase in the 2D frequency domain. The range of $f_{\eta }$ is [−10,000, 10,000] Hz.

**Figure 5:**
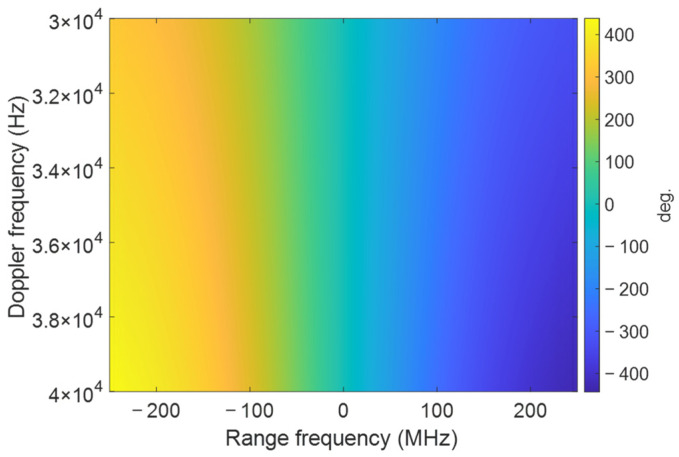
The compensation phase in the 2D frequency domain with a non-zero Doppler frequency. The range of $f_{\eta }$ is [30,000, 40,000] Hz.

For the signal design in Section 2.3, suppose the range of Doppler frequency $f_{\eta }$ is [−10,000, 10,000] Hz; the compensation phase can be seen in Figure 4. It can be found that the error is symmetric along the center point (0,0), where the error at the edge can be up to ±100 degrees, and the relative error can be up to 200 degrees, which is large enough to cause target defocusing. Suppose the range of Doppler frequency $f_{\eta }$ is [30,000, 40,000] in the presence of a squint angle; then, the compensation phase can be seen in Figure 5. At this point, the error at the edge can be ±400 degrees. Such a large error makes the target seriously defocused.

### 4.2. Proposed Compensation Method

**Figure 6:**
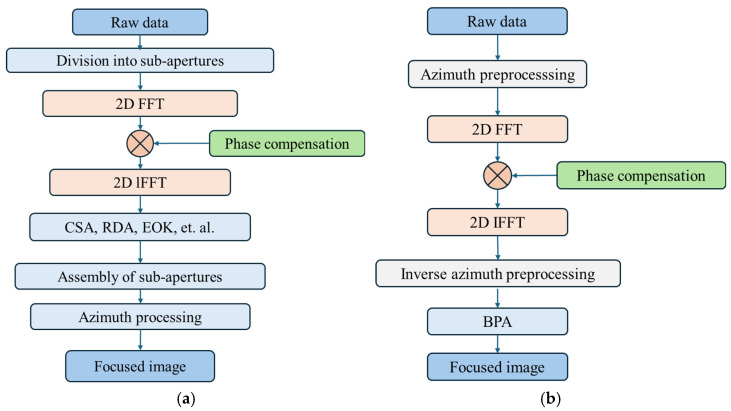
The flowchart of the proposed compensation method. (**a**) Integrating with the algorithm proposed in [5]; (**b**) Integrating with the algorithm proposed in [7].

The developed phase compensation framework demonstrates operational compatibility with two established SAR imaging architectures: When synergized with the sub-aperture processing scheme in [5] (Figure 6a), the workflow initiates with raw data segmentation into multiple sub-apertures, followed by 2D Fourier transformation of each segment into the 2D spectral domain where phase correction $\theta_{error}$ is applied, after which conventional reconstruction algorithms generate sub-images that are subsequently aggregated through coherent synthesis and azimuth focusing to yield final image with full resolution. Alternatively, when integrated with the hybrid backprojection architecture from [7] (Figure 6b), azimuth preprocessing first resamples raw data in the Doppler domain, enabling joint 2D spectral compensation prior to inverse preprocessing operations, ultimately culminating in precision imaging via backprojection algorithms. The complexity of the proposed method is the same as [5] and [7], respectively.

## 5. Simulation Experiments

**Figure 7:**
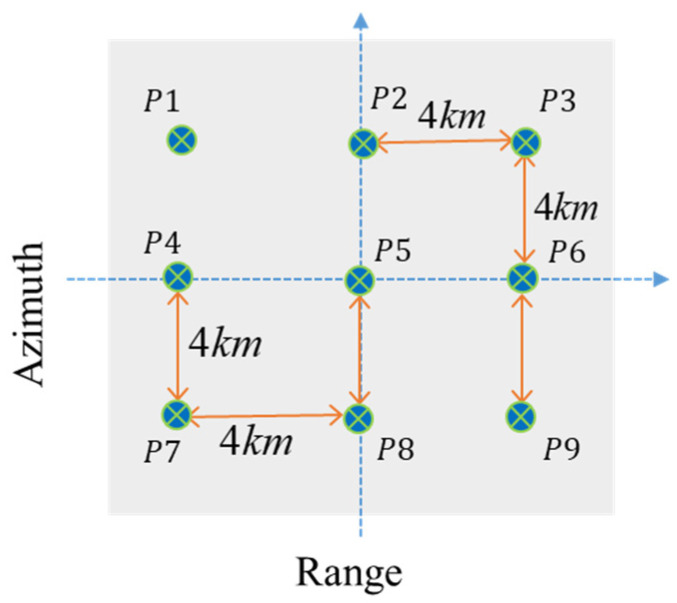
Point target array in the simulation scene.

In this section, simulation experiments are performed to verify the effectiveness of the proposed compensation method. The simulation experiments are conducted using the parameters listed in Table 2. The satellite operates at an orbit height of 500 km with a carrier frequency of 10 GHz and an incidence angle of 30°. The range of bandwidth and sampling frequency are set to 500 MHz and 600 MHz, respectively. In addition, the pulse duration and pulse repetition frequency (PRF) are configured as 60 μs and 5000 Hz, respectively. The synthetic aperture time is 6.55 s. A 3 × 3 point target array is arranged in an 8 km × 8 km scene, as illustrated in Figure 7.

**Figure 8:**
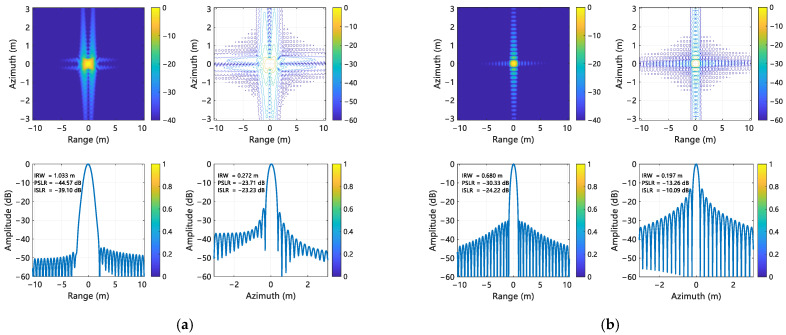
Imaging results of P1. (**a**) Without error compensation; (**b**) With error compensation.

**Figure 9:**
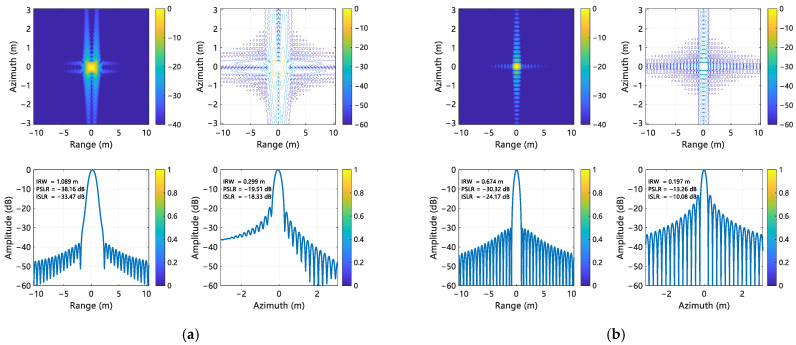
Imaging results of P5. (**a**) Without error compensation; (**b**)With error compensation.

**Figure 10:**
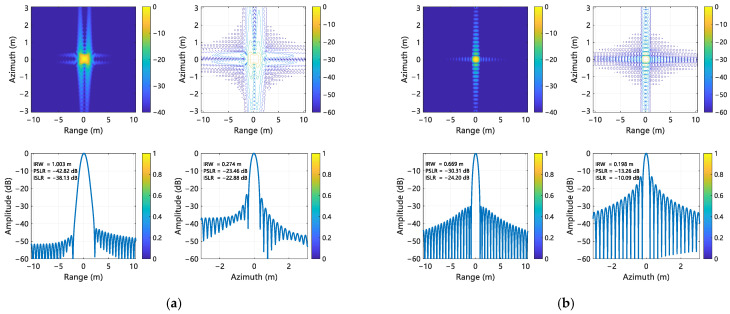
Imaging results of P9. (**a**) Without error compensation; (**b**) With error compensation.

For range processing, a Taylor window with four quasi-equiripple sidelobes adjacent to the main lobe was applied, constraining peak sidelobe magnitudes to −30 dB relative to the mainlobe. The focusing performance of the proposed compensation method was evaluated using point targets P1, P5, and P9, located at the corners and center of the scene. The imaging results for these targets are shown in Figure 8, Figure 9, and Figure 10, respectively. Quantitative metrics, including IRW, PSLR, and integrated sidelobe ratio (ISLR) in both range and azimuth directions, are summarized in Table 3 and Table 4. A comparison and analysis of imaging results is hereby presented, utilizing Target 5 as an example. It is found that the range IRW is up to 1.089 m when the error is not corrected, and the range IRW is 0.674 m after the error is corrected. It can thus be seen that the IRW deteriorates by 62% when the error exists. A similar trend is observed when comparing the azimuth IRW, which deteriorates by 52% when an error exists. Following correction, the IRW is 0.197 m, indicating a recovery in performance. Furthermore, the range towards PSLR and ISLR were −38.16 dB and −33.47 dB, respectively, when the error existed, which was inconsistent with the metrics designed at the beginning. However, subsequent to the implementation of error correction, the values were found to be −30.32 dB and −24.17 dB, respectively, thereby aligning with the pre-defined metrics. This outcome serves to corroborate the efficacy of the implementation of the error correction process.

The comparison results indicate that the absence of error compensation results in suboptimal performance in terms of distance and azimuthal focusing quality, accompanied by target defocus. Conversely, the implementation of the proposed algorithm enhances the range and azimuth focusing quality, thereby substantiating the efficacy of the proposed algorithm.

## 6. Conclusions

NLFM signals have been demonstrated to significantly enhance the image quality of SAR systems with optimized waveform time-frequency functions. This has led to significant potential for NLFM signals within the domain of SAR systems. However, significant errors introduced by the simplified start–stop model in SAR systems using NLFM signals cause target defocus, which degrades the image quality. In this paper, a general numerical error compensation method for NLFM signal in SAR system based on the non-start–stop model is proposed. The error is derived from the non-start–stop model and numerical computation compensation. In addition, simulation experiments are carried out to demonstrate the effectiveness of the proposed method. The results show that the proposed method can compensate for phase errors and obtain well-focused targets, which has important application potential in future SAR development. Future work will focus on exploring its application in multichannel and MIMO SAR systems.

## Figures and Tables

**Table 1:** Simulation parameters of NLFM signal (Taylor window).

Parameter	Value
Range bandwidth	500 MHz
Carrier frequency	10 GHz
Pulse duration	60 μs
Sidelobe number	4
Relative to the mainlobe.	−30 dB

**Table 2:** Simulation parameters.

Parameter	Value
Orbit height	500 km
Carrier frequency	10 GHz
Incidence angle	30°
Range bandwidth	500 MHz
Range sampling frequency	600 MHz
Pulse duration	60 μs
PRF	5000 Hz
Acquisition time	6.55 s

**Table 3:** IRW, PSLR, and ISLR of target P1–P9 without error compensation.

Targets	Range			Azimuth		
	IRW (m)	PSLR (dB)	ISLR (dB)	IRW (m)	PSLR (dB)	ISLR (dB)
P1	1.033	−44.57	−39.10	0.272	−23.71	−23.23
P2	1.013	−43.17	−38.41	0.272	−23.15	−22.42
P3	1.068	−38.82	−34.82	0.292	−20.69	−19.58
P4	1.018	−47.45	−40.60	0.270	−24.00	−23.63
P5	1.089	−38.16	−33.47	0.299	−19.51	−18.33
P6	0.998	−48.17	−41.15	0.270	−24.07	−23.74
P7	1.081	−38.65	−34.37	0.294	−20.40	−19.36
P8	1.229	−34.03	−29.57	0.354	−16.23	−15.06
P9	1.003	−42.82	−38.13	0.274	−23.46	−22.88

**Table 4:** IRW, PSLR, and ISLR of target P1–P9 with error compensation.

Targets	Range			Azimuth		
	IRW (m)	PSLR (dB)	ISLR (dB)	IRW (m)	PSLR (dB)	ISLR (dB)
P1	0.680	−30.33	−24.22	0.197	−13.26	−10.09
P2	0.675	−30.32	−24.21	0.197	−13.26	−10.09
P3	0.669	−30.34	−24.20	0.198	−13.26	−10.09
P4	0.680	−30.32	−24.18	0.197	−13.26	−10.09
P5	0.674	−30.32	−24.17	0.197	−13.26	−10.08
P6	0.669	−30.30	−24.16	0.198	−13.26	−10.09
P7	0.680	−30.29	−24.22	0.197	−13.26	−10.09
P8	0.675	−30.29	−24.21	0.197	−13.26	−10.09
P9	0.669	−30.31	−24.20	0.198	−13.26	−10.09

## Data Availability

The original contributions presented in the study are included in the article. Further inquiries can be directed to the corresponding author.

## Abbreviations

The following abbreviations are used in this manuscript:

SAR	synthetic aperture radar
LFM	linear frequency modulated
NLFM	nonlinear frequency modulated
IRW	impulse response width
PSLR	peak sidelobe ratio
ISLR	integrated sidelobe ratio
POSP	principle of stationary phase
PSD	power spectral density
SNR	signal-to-noise ratio
PRI	pulse repetition interval
PRF	pulse repetition frequency
MIMO	multipe input multiple output
